# Circulating biomarkers associated with pyroptosis in the differential diagnosis of ST-segment elevation and non-ST-segment elevation myocardial infarction

**DOI:** 10.3389/fcvm.2025.1648568

**Published:** 2025-11-25

**Authors:** Zeki Dogan, Abdulhalim Senyigit, Seyma Dumur, Naile Fevziye Misirlioglu, Nedim Uzun, Sumeyye Nur Aydin, Adem Melekoglu, Ertugrul Altinbilek, Ufuk Çakatay, Hafize Uzun

**Affiliations:** 1 Department of Cardiology, Faculty of Medicine, Istanbul Atlas University, Istanbul, Türkiye; 2 Department of Internal Medicine, Faculty of Medicine, Istanbul Atlas University, Istanbul, Türkiye; 3 Department of Biochemistry, Faculty of Medicine, Istanbul Atlas University, Istanbul, Türkiye; 4 Department of Emergency, Gaziosmanpaşa Training and Research Hospital, University of Health Sciences, Istanbul, Türkiye; 5 Department of Public Health, Istanbul Provincial Health Directorate, Istanbul, Türkiye; 6 Department of Emergency, Sisli Hamidiye Etfal Education and Research Hospital, University of Health Sciences, Istanbul, Türkiye; 7 Department of Medical Biochemistry, Cerrahpasa Faculty of Medicine, Istanbul University-Cerrahpasa, Istanbul, Türkiye

**Keywords:** caspase-1, IL-1β, IL-18, myocardial infarction, ST-segment elevation, pyroptosis

## Abstract

**Objectives:**

To evaluate the biomarker value of NOD-, LRR-, and pyrin domain-containing protein 3 (NLRP3), gasdermin D (GSDMD), caspase-1, interleukin 1β (IL-1β), and IL-18 in the systemic circulation in ST-elevation myocardial infarction (STEMI) and non-STEMI (NSTEMI) as a possible new biomarker candidate, and to compare their sensitivity and specificity to well-defined and built in biomarkers in acute myocardial infarction (AMI), including high sensitive-cardiac troponin I (hs-cTnI) and N-terminal proBNP (NT-proBNP) in patients with STEMI or NSTEMI.

**Methods:**

The study included 149 patients with acute STEMI, 151 patients with NSTEMI, and 151 healthy volunteers in the check-up outpatient clinic, admitted to Sisli Hamidiye Etfal Education and Research Hospital, Emergency Department, Istanbul.

**Results:**

CK-MB, hs-cTnI, pro-BNP, NLRP3, GSDMD, caspase-1, IL-1β, and IL-18 levels were higher in the STEMI group compared to the other groups, whereas these parameters were lower in the control group compared to the NSTEMI groups. hs-cTnI, CK-MB, caspase-1, IL-1β, and IL-18 showed high sensitivity and specificity, suggesting their potential as reliable diagnostic markers in STEMI patients.

**Conclusions:**

NLRP3 inflammasome may play a role in the production of pro-inflammatory cytokines such as IL-1β and IL-18 in STEMI. Inflammasome-related biomarkers show potential as adjunctive tools for distinguishing between STEMI and NSTEMI; however, further studies with multiple timepoints and larger sample sizes are needed to confirm their diagnostic utility and clinical applicability.

## Introduction

The most common cardiovascular disease (CVD), acute coronary syndrome (ACS), is the leading global cause of morbidity and mortality. The term acute myocardial infarction (AMI) is used when there is evidence of myocardial necrosis because of ischemic injury. Traditional AMI classification distinguishes ST-segment elevation myocardial infarction (STEMI) from non-ST-segment height myocardial infarction (NSTEMI), depending on the presence or absence of permanent ST-segment elevation in the electrocardiogram. STEMI continues to be the leading cause of sudden death worldwide, and despite recent developments, there is continued discussion in the literature about its optimal clinical management (1).

Pyroptosis is an inflammatory form of programmed cell death triggered by inflammasome activation, leading to the release of IL-1β, IL-18, and damage-associated molecular patterns (DAMPs). Gasdermin D (GSDMD) is essential for this process, forming membrane pores that release cytokines. Caspase-1, activated by the NLRP3 inflammasome, cleaves pro-IL-1β and pro-IL-18 into their active forms. This pathway plays a crucial role in atherosclerosis and other inflammatory diseases (2–4). The activation of the NLRP3 inflammasome, with subsequent release of pro-inflammatory cytokines like IL-1β and IL-18, is a critical mechanism in the pathogenesis of CVD. Key molecules like NLRP3, GSDMD, caspase-1, IL-1β, and IL-18 are involved in driving inflammation, cell death, and tissue damage. Targeting these pathways represents a promising approach to developing new therapies for inflammatory cardiovascular conditions, with several clinical trials investigating the efficacy of inflammasome inhibitors in CVD management (4).

Pyroptosis, a form of programmed inflammatory cell death mediated by the NLRP3 inflammasome, has recently emerged as a critical mechanism in the pathogenesis of atherosclerosis and plaque rupture. Upon activation, the NLRP3 inflammasome promotes the release of pro-inflammatory cytokines such as IL-1β and IL-18 through caspase-1 and the pore-forming protein GSDMD. However, the diagnostic utility of these pyroptosis-associated biomarkers in a clinical AMI setting, particularly in distinguishing between STEMI and NSTEMI, has not been well characterized. Although evidence from preclinical models has demonstrated the role of inflammasome activation in cardiovascular tissue damage, translating these findings into clinical practice remains a challenge. Therefore, this study aims to evaluate the clinical diagnostic value of circulating pyroptosis-related biomarkers (NLRP3, GSDMD, caspase-1, IL-1β, and IL-18) in patients with STEMI and NSTEMI, and to compare their sensitivity and specificity with standard cardiac biomarkers (hs-cTnI and NT-proBNP).

## Research design and methods

### Ethical considerations

This study was conducted according to the guidelines of the Declaration of Helsinki and approved by the Sisli Hamidiye Etfal Education and Research Hospital, University of Health Sciences Clinical Research Ethics Committee (file number of approval: 2461; Date: 10.10.2023). All subjects gave informed consent for inclusion before participating in the study.

### Study design and patient groups

The study included 149 patients with acute STEMI and 151 patients with NSTEMI admitted to Sisli Hamidiye Etfal Education and Research Hospital, Emergency Department, and Medicine Hospital, Cardiology Department, between November 2023 and June 2024. After admission, vital signs were recorded, and a physical examination was performed.

**Figure 1:**
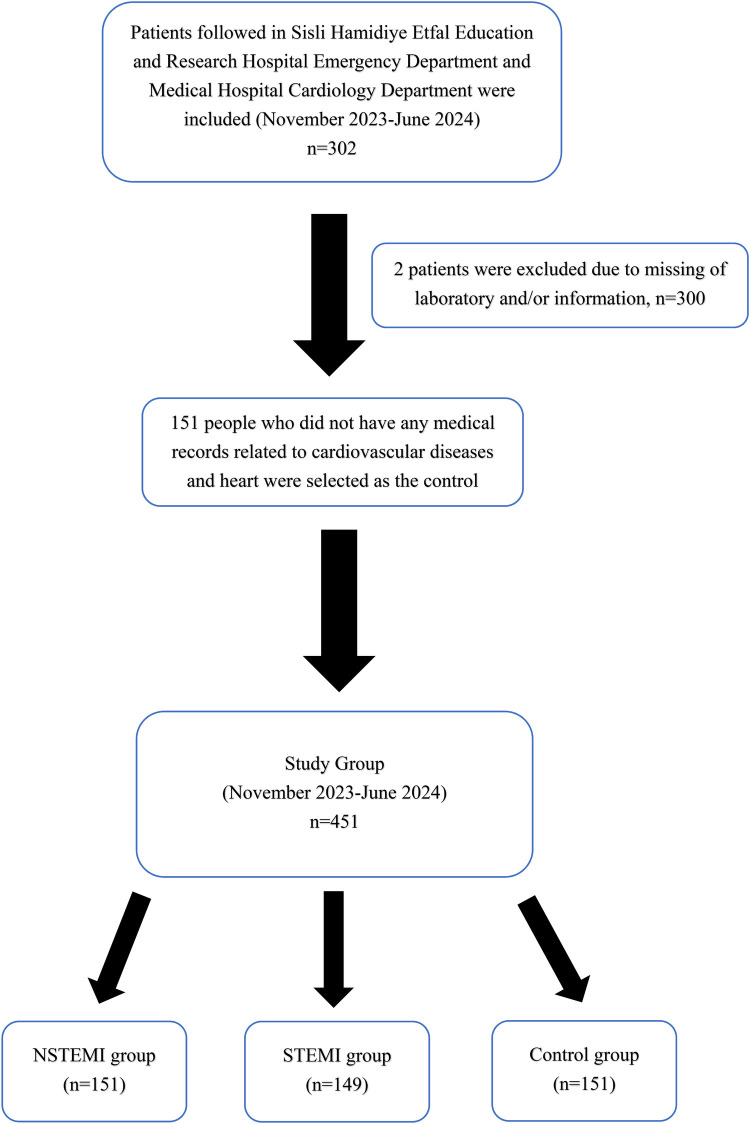
A flowchart of the selection of cases.

The control group comprised 151 subjects presenting to the check-up outpatient clinic at Sisli Hamidiye Etfal Education and Research Hospital. Control subjects were free of known cardiovascular disease, chronic inflammatory or autoimmune disorders, active infections, and malignancy, and were not using medications known to influence inflammatory markers (e.g., NSAIDs, corticosteroids) (Figure 1).

The American Heart Association, American College of Cardiology, European Society of Cardiology, and World Heart Federation have established guidelines (5). Evaluating patients with an acute onset of chest pain should begin with an ECG and troponin levels. The American College of Cardiology (ACC), American Heart Association (AHA), ESC, and the World Heart Federation (WHF) committee established the following ECG criteria for STEMI:

New ST-segment elevation occurs at the J point in 2 contiguous leads, with a threshold greater than 0.1 mV in all leads except V2 and V3.

In leads V2 and V3, the threshold is greater than 0.2 mV for men older than 40, greater than 0.25 mV for men under 40, and greater than 0.15 mV for women.

### Inclusion criteria

The diagnosis of STEMI is based on the presence of at least one of the following specific diagnostic criteria for the diagnosis of AMI, together with an increase and/or decrease in cardiac biomarkers (preferably troponin) such that at least one value exceeds the 99th percentile of the upper reference limit:

**Table:** 

(i) Patients with first AMI
(ii) Acute typical ischemic chest pain
(iii) New or presumably new significant ST-T changes (ST-segment elevation measured in at least two consecutive leads from point J on ECG with standard calibration of 25 mm/sc and 10 mm/mV in leads V2 -V3 is ≥2.5 mm in leads V2–V3, ≥2 mm in leads 40 years of age and older, ≥1.5 mm in women or ≥1 mm in other leads) or new Left Bundle Branch Block
(iv) Development of pathological Q waves on ECG was defined as. NSTEMI was diagnosed when the patient complained of acute chest pain with elevated cTn-I values (>1.0 ng/ml in any sample within the first 9 h after admission) with or without ST/T changes on electrocardiogram (ECG), and no other apparent cause for this chest pain

### Exclusion criteria

**Table:** 

(i) Patients with clinical conditions such as acute renal failure, chronic renal failure (CRF), cerebrovascular disease, diabetic ketoacidosis, pulmonary edema, acute abdomen, sepsis, trauma, and trauma patients were not included in the study
(ii) Patients with a previous diagnosis of advanced aortic stenosis, decompensated heart failure, ventricular tachycardia, hepatic dysfunction, acute pericarditis or myocarditis, skeletal muscle disease, systemic infection, and cancer patients were also excluded

### Assessments of the electrocardiogram (ECG)

The standard 12-lead ECG has been performed for various indications, including emergency department inpatient beds and outpatient facilities. ECGs were taken within 10 min after admission to the hospital, and ECG results were obtained from the patient's file. Patients were classified as STEMI and NSTEMI according to ECG findings. In patients without typical ST wave changes and Q waves in the first hours of infarction, ECGs were repeated and compared with the first ECG. STEMI and NSTEMI were performed according to the ESC 2023 guidelines (6).

### Blood collection and processing

Blood samples were taken within the first 2 h from STMI and NSTEMI patients who met the above criteria and presented to the emergency department at any time. Blood samples were drawn from the brachial veins in the brachial fossa and placed into anticoagulant-free tubes and K3 EDTA tubes. The samples were centrifuged for 10 min at 4,000 rpm at 4°C. Routine clinical chemistry analyses were performed immediately. To determine other analytical parameters, serum aliquots were frozen and stored at −80°C until they were required for further analysis.

The routine clinical chemistry parameters were assessed with an automated biochemistry analyzer (Architect i2000, Abbott Park, IL, USA). The serum highly sensitive C-reactive protein (hsCRP) levels were measured using the nephelometric method (Immage 800 Beckman Coulter, CA 92821, USA). The highly sensitive cTnI (hs-cTnI) levels were assessed by the immunofluorescent method using fluorescent antibody conjugates (ARCHITECTR Abbott assay, USA). The N-terminal proBNP (NT-proBNP) levels were assayed by the chemiluminescence method using an I2000 architect machine (ARCHITECTR, Abbott, USA).

NLRP3, GSDMD, caspase-1, IL-1β, and IL-18 levels were assessed using commercial ELISA kits from BT LAB, Zhejiang, China. Intra- and inter-assay variations for all assays were below 8.8% and 10.0%, respectively, ensuring reliable measurements.

### Sample size and power analysis

A total of 451 patients were included in the study (STEMI, *n* = 149; NSTEMI, *n* = 151; control group, *n* = 151). A *post-hoc* power analysis was performed based on this sample size. An alpha error level of 0.05, the overall statistical power of the study was estimated to exceed 80%; this indicates that the sample size was sufficient to detect observed group differences with adequate reliability.

### Statistical analysis

All statistical analyses were performed with SPSS version 26 software and MedCalc statistical software. The compatibility of the variables with normal distribution was examined by the Shapiro–Wilk test and Q-Q plot, and histogram graphs. As a result of the analysis, normally distributed variables were shown as mean ± standard deviation and non-normally distributed variables as median (minimum-maximum). Categorical data were shown as frequency (percentage). Categorical variables were evaluated by the Chi-Squared Test or Fisher's Exact test. In comparisons between independent groups, the Student *T* Test was used between two groups when the distribution of data was normal, and the Way Anova test was used in more than two groups. When the distribution of the data was not normal, the Mann–Whitney *U* Test was used for two-group comparisons, and the Kruskal–Wallis test was used for more than two-group comparisons. The relationship between numerical variables was evaluated using the Spearman correlation test. The sensitivity and specificity of the measured variables for biomarkers were examined using a receiver operating characteristic (ROC) curve analysis. Confidence intervals were calculated using the Bootstrap method [Bootstrap method performs multiple resampling (bootstrapping) to evaluate the performance of the model, calculates AUROC for each sample, and then gives confidence intervals]. The area under the curve (AUC) was calculated for each biomarker. To assess whether there were significant differences in diagnostic accuracy between biomarkers, the AUC values were compared using the pairwise DeLong test. The DeLong test is a non-parametric method used to statistically compare the AUC differences between dependent sample ROC curves. In multivariate analyses, independent predictors of hs-cTnI level change were examined using linear regression. Potential predictors identified in preliminary analyses, including CK-MB, Gasdermin D, Caspase-1, and IL-18, were entered into the model. Both Enter and Forward stepwise methods were applied to evaluate the robustness of the results. Logistic regression models were established to evaluate the discriminatory role of inflammasome markers between the STEMI and NSTEMI subgroups. STEMI vs. NSTEMI was defined as the dependent variable. All models were adjusted for fasting glucose, HbA1c, and smoking status to control for potential metabolic and lifestyle confounders. A separate model was created for each inflammasome marker (NLRP3, IL-1β, Caspase-1, and Gasdermin D), and results were reported as odds ratios (OR) with 95% confidence intervals (CI). The Hosmer-Lemeshow test was used for model fit. Model fit was analyzed using the required residual and fit statistics. For all analyses, *p* < 0.05 was considered significant.

## Results

The differences in demographic and clinical characteristics between the groups were evaluated. The systolic blood pressure (SBP) and diastolic blood pressure (DBP) of the patients in the NSTEMI group were significantly higher compared to the control group (*p* < 0.001; *p* < 0.001). There was no difference between the SBP of the patients in the NSTEMI and STEMI groups. The pulse rate of the patients in the STEMI group was significantly higher compared to the control group and the NSTEMI group (*p* < 0.001). There was no difference in the pulse rate of the patients in the NSTEMI and control groups. It was observed that cigarette use was higher in the STEMI group compared to the control group. There was no significant difference between the groups in terms of alcohol use. DM and HT were observed to be higher in patients in the STEMI group compared to the NSTEMI group. The difference between the study groups is presented in Table 1.

**Table 1:** Evaluation of the differences in demographic and clinical characteristics between the study groups.

Characteristics	Control group (*n* = 151)	NSTEMI group (*n* = 151)	STEMI group (*n* = 149)	*p* value
Gender (Female/Male) (%)	44/56	42/58	56/44	**0**.**022**^a^
Age (year)	65.0 (54–78)	64 (52–75)	64.0 (49–75.5)	0.268^b^
Body mass index (BMI) (kg/m^2^)	24.4 (23.2–25.2)*	25.2 (23.7–28.1)*	27.7 (25.9–28.7)*	**<0**.**001**^b^
Systolic blood pressure (SBP) (mmHg)	125.0 (120–130)^d^	130.0 (120–150)^c^	132.0 (120–144)^c^	**<0**.**001**^b^
Diastolic blood pressure (DBP) (mmHg)	74.0 (68–75)	85.0 (78–86)	75.0 (68–80)	**<0**.**001**^b^
Pulse (beats per minute)	78.0 (72–80)	77.0 (75–80)	80.0 (77–80)^c^, ^d^	**<0**.**001**^b^
Smoking *n* (%)	0 (%0.0)	30 (%19.9)	106 (%71.1)	**<0**.**001**^a^
Alcohol *n* (%)	0 (%0.0)	32 (%21.2)	26 (%17.4)	0.412^a^
Diabetes Mellitus (DM) *n* (%)	0 (%0.0)	25 (%16.6)	93 (%62.4)	**<0**.**001**^a^
Hypertension (HT) *n* (%)	0 (%0.0)	68 (%45.0)	85 (%57.0)	**0**.**037**^a^
Cardiovascular complications *n* (%)	0 (%0.0)	12 (%7.9)	20 (%13.4)	0.124^a^
Renal complications *n* (%)	0 (%0.0)	27 (%17.9)	24 (%16.1)	0.683^a^
Hepatic complications *n* (%)	0 (%0.0)	0 (%0.0)	1 (%0.7)	0.497^a^

Bold values indicate statistically significant differences (*p* < 0.05).

*Median (25.–75. Percentile).

a Pearson Chi Square Test.

b Kruskall Wallis Test.

c Refers to the difference with the control group.

d Refers to the difference with the NSTEMI group.

In the *post hoc* analyses, fasting blood glucose levels were higher in the STEMI group compared to the other groups; the difference between the control and NSTEMI groups was not significant. HbA1c was higher in the STEMI group compared to the other groups. Cholesterol levels were higher in the NSTEMI group compared to the other groups; the difference in the cholesterol levels between the control group and the STEMI group was not significant. After adjustment for multiple comparisons, biochemical and inflammasome markers remained statistically significant, as confirmed by both Bonferroni and Benjamini–Hochberg false discovery rate (FDR) corrections (Table 2).

**Table 2:** Evaluation of the difference between the biochemistry levels and pyroptosis biomarkers of the study groups.

Parameters	Control (*n* = 151)	NSTEMI (*n* = 151)	STEMI (*n* = 149)	*p* value
Fasting blood glucose (mg/dl)	93.0 (86–96)	89.0 (85–100)	110.0 (92–146)^a^^,^^b^	**<0**.**001**
HbA1c (%)	5.2 (5.1–5.8)^b^	5.7 (5.1–5.8)^a^	7.6 (5.8–8.7)^a^^,^^b^	**<0**.**001**
Total Cholesterol (mg/dl)	145.0 (142–163)^b^	180.0 (150–202)^a^	169.0 (141–189)^b^	**<0**.**001**
LDL-C (mg/dl)	87.0 (69–102)^b^	96.0 (80–112)^a^	102.0 (82–124)^a^	**0**.**009**
HDL-C (mg/dl)	36.0 (35–45)^b^	45.0 (42–56)^a^	37.0 (30.5–44.0)^b^	**<0**.**001**
Triglyceride (mg/dl)	96.0 (69–114)^b^	128.0 (105–150)^a^	1,119.0 (96–155)^a^	**<0**.**001**
Total protein (g/dl)	68.0 (66–70)	68.0 (66–74.5)	68.6 (63–75)	0.611
Albumin (g/dl)	39.4 (36–40.4)^b^	40.5 (36–42)^a^	39.8 (37–41)^a^^,^^b^	**0**.**001**
Urea (mg/dl)	36.0 (25–42)	47.0 (38–61)^a^	37.0 (31–56)^a^^,^^b^	**<0**.**001**
Creatinine (mg/dl)	0.9 (0.7–1.0)^b^	0.9 (0.8–1.1)^a^	1.1 (0.8–1.2)	**0**.**009**
Uric acid (mg/dl)	4.1 (3.5–4.7)	3.9 (3.6–5.4)	3.8 (3.5–4.9)	0.377
AST (U/L)	25.0 (18–30)^b^	29.0 (25–41)^a^	26.0 (17–34)^b^	**<0**.**001**
ALT (U/L)	16.0 (13–26)	15.0 (15–31)	17.0 (15–23)	0.284
LDH (U/L)	156.0 (144–187)^b^	251.0 (207–319)^a^	200 (154–255)^a^^,^^b^	**<0**.**001**
CK-MB (ng/ml)	12.6 (10.5–16.5)^b^	82.3 (23.8–91.6)^a^	255 (154–533)^a^^,^^b^	**<0**.**001**
hs-cTnI (ng/ml)	0.05 (0.04–0.07)^b^	1.8 (0.1–2.6)^a^	35.5 (23–43)^a^^,^^b^	**<0**.**001**
NT-proBNP (pg/ml)	74.0 (59–96)^b^	1,022 (963–1,152)^a^	2,100 (1,926–2,869)^a^^,^^b^	**<0**.**001**
CRP (mg/L)	3.0 (2.5–4.1)^b^	16.5 (3.8–22.6) ^a^	12.0 (2.0–47.0) ^a^^,^^b^	**<0**.**001**
NLRP3 (pg/ml)	42.3 (36.4–51.0)^b^	91.6 (87.6–93.4)^a^	128.4 (125–141)^a^^,^^b^	<0.001
Gasdermin D (ng/ml)	3.6 (2.3–6.0)^b^	14.2 (13.0–17.4)^a^	17.5 (14.5–19.1)^a^^,^^b^	<0.001
Caspase 1 (ng/ml)	9.6 (8.0–12.7)^b^	15.0 (14–15.9)^a^	19.4 (18.2–20.1)^a^^,^^b^	<0.001
IL-1βeta (ng/L)	11.0 (9.0–14.2)^b^	23.8 (21–26)^a^	32.0 (28.7–33.1)^a^^,^^b^	<0.001
IL-18 (ng/L)	13.1(12.1–17.0)^b^	23.8(22–26)^a^	38.2(36.5–42.1)^a^^,^^b^	<0.001

Kruskall Wallis Test.

LDL-C, low-density lipoprotein cholesterol; LDL-C, high-density lipoprotein cholesterol; AST, aspartate aminotransferase; ALT, alanine aminotransferase; LDH, lactate dehydrogenase; CK-MB, creatine kinase-MB; hs-cTnI, Troponin-I; NT-proBNP, N-terminal proBNP; CRP, C-reactive protein; NLRP3, NOD-, LRR-, and pyrin domain-containing protein 3; IL, interleukin.

Values in bold denote statistically significant differences at the 0.05 level (*p* < 0.05).

a Refers to the difference with the control group.

b Refers to the difference with the NSTEMI group.

The pyroptosis biomarkers in the study groups are presented the Table 3. The difference between the CK-MB, hs-cTnI, pro-BNP, NLRP3, GSDMD, caspase-1, IL-1β, and IL-18 levels of the study groups was evaluated. Statistically significant differences were observed between the parameters, and *post hoc* analyses showed that there was a difference between the two groups in all parameters. CK-MB, hs-TnI, pro-BNP, NLRP3, GSDMD, caspase-1, IL-1β, and IL-18 levels were higher in the STEMI group compared to the other groups, whereas these parameters were lower in the control group compared to the NSTEMI groups. Multivariable analyses demonstrated that the associations between inflammasome markers and STEMI remained significant after adjustment for fasting glucose, HbA1c, and smoking status. Subgroup analyses within STEMI patients stratified by diabetes, poor glucose control, and smoking history revealed similar trends, suggesting that the observed inflammasome activation in STEMI is not solely attributable to metabolic disturbances or smoking.

**Table 3:** Logistic regression analysis for the association between inflammasome markers and STEMI (vs. NSTEMI).

Parameters	OR (95% CI)	*p*-value
NLRP3	2.11 (1.03–4.32)	**0**.**041**
IL-1β	2.69 (1.97–3.68)	**<0**.**001**
Caspase-1	32.38 (6.02–174.1)	**<0**.**001**
Gasdermin D	1.51 (1.32–1.74)	**<0**.**001**

IL-18 values showed near-complete separation between STEMI and NSTEMI groups, preventing stable estimation by standard logistic regression.

Model fit indices (STEMI vs. NSTEMI adjusted models): Hosmer–Lemeshow *χ*^2^ (df) = 5.6–7.9, *p* > 0.40; Nagelkerke *R*^2^ = 0.19–0.32; −2 Log Likelihood = 198.5–225.3.

Logistic regression analysis, models adjusted for fasting glucose, HbA1c, and smoking status.

Values in bold denote statistically significant differences at the 0.05 level (*p* < 0.05).

Adjusted analyses confirmed that IL-1β, gasdermin D, caspase-1, and NLRP3 were independently elevated in STEMI compared with NSTEMI, independent of fasting glucose, HbA1c, and smoking status (Table 3).

**Figure 2:**
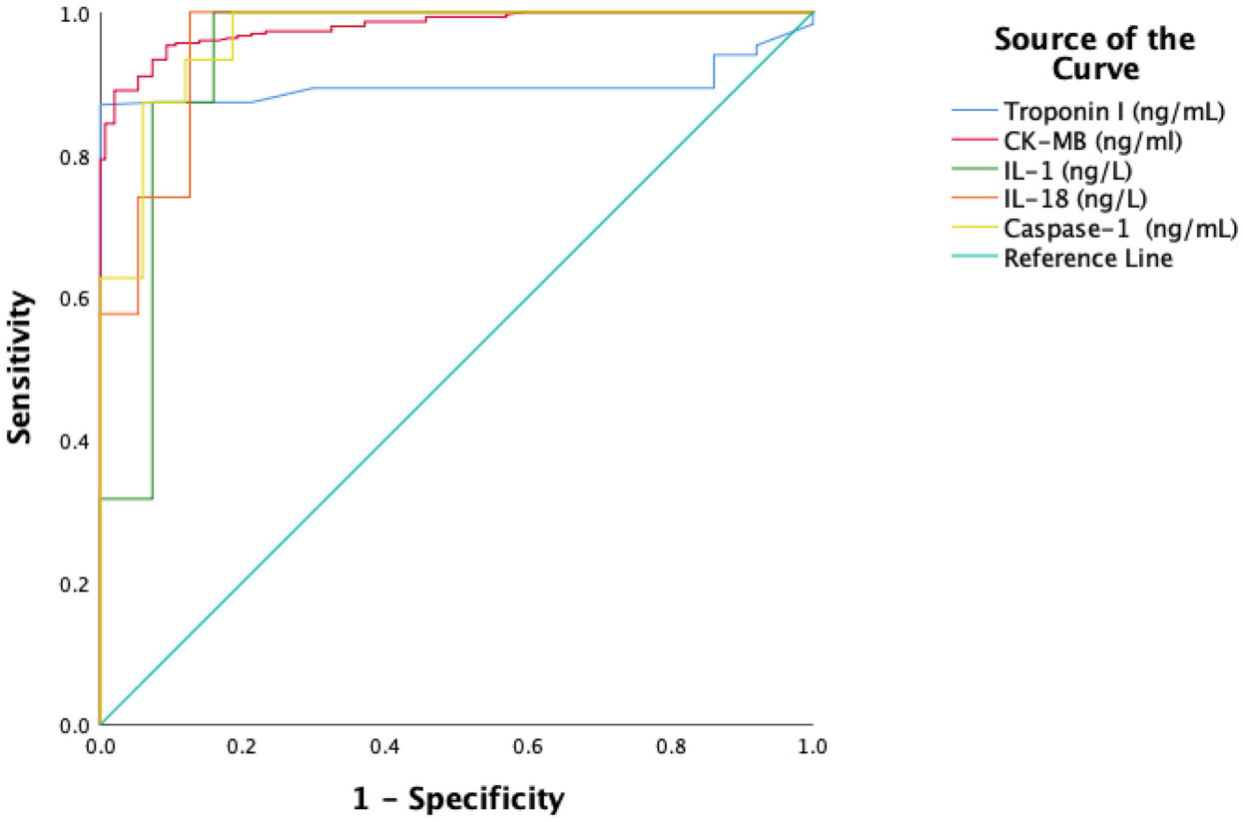
Comparison of ROC curves of clinical parameters used in the diagnosis of acute myocardial infarction.

In Figure 2, the power of clinical parameters to indicate the risk of AMI is compared in ROC curves. The optimal cut-off value of −0.09 achieved 87% sensitivity and 92% specificity, making hs-cTnI a highly reliable marker for diagnosis and monitoring in STEMI patients, while for CK-MB, a cut-off value of 21.9 resulted in 91% sensitivity and 94% specificity. For IL-1β, a cut-off value of 20.6 resulted in 93% sensitivity and 84% specificity, while IL-18 had a cut-off value of 20.8, achieving 94% sensitivity and 87% specificity. For caspase-1, a cut-off value of 13 resulted in 96% sensitivity and 81% specificity (Table 4). According to the ROC analysis, the diagnostic accuracy of CK-MB (AUC = 0.979), caspase-1 (AUC = 0.966), IL-1β (AUC = 0.939), and IL-18 (AUC = 0.959) biomarkers was higher compared to hs-cTnI (AUC = 0.897). The results of the pairwise DeLong test indicate significant differences between hs-cTnI and caspase-1 (*p* < 0.001), IL-18 (*p* < 0.008), and CK-MB (*p* < 0.001). The difference between hs-cTnI and IL-1β was not significant (*p* = 0.051). No significant differences were observed between other biomarker pairs: caspase-1-IL-1 (*p* = 0.105), caspase-1-IL-18 (*p* = 0.570), IL-1β -IL-18 (*p* = 0.286), caspase-1-CK-MB (*p* = 0.162), and CK-MB-IL-18 (*p* = 0.053). However, CK-MB- IL-1β showed a significant difference (*p* = 0.010) (Table 5).

**Table 4:** Evaluation of AUC and optimum cut-off point of clinical parameters.

Parameters	AUC (%95 CI)	*p* value	Cut-off	Sensitivity (%)	Specificity (%)
hs-cTnI (ng/ml)	0.897 (0.864–0.929)	<0.001	0.09	**87**	**92**
CK-MB (ng/ml)	0.979 (0.969–0.989)	<0.001	21.9	**91**	**94**
Caspase-1 (ng/ml)	0.966 (0.950–0.982)	<0.001	13.0	**96**	**81**
IL-1β (ng/L)	0.939 (0.910–0.968)	<0.001	20.6	**93**	**84**
IL-18 (ng/L)	0.959 (0.940–0.978)	<0.001	20.8	**94**	**87**

CK-MB, creatine kinase-MB; IL, interleukin.

Bold values indicate parameters with the highest sensitivity and specificity at the optimum cut-off point.

Confidence intervals were calculated using the Bootstrap method.

**Table 5:** Evaluation of the significant difference in diagnostic accuracy between parameters.

Parameter 1	Parameter 2	AUC 1	AUC 2	Difference between areas (%95 CI)	*Z* statistic	DeLong *p* value
hs-cTnI	CK-MB	0.897	0.979	0.08 (0.04–0.11)	4, 963	**<0**.**001**
hs-cTnI	Caspase-1	0.897	0.966	0.06 (0.03–0.104)	3, 879	**<0**.**001**
hs-cTnI	IL-1β	0.897	0.939	0.04 (−0.0, 003–0.08)	1, 944	<0.051
hs-cTnI	IL-18	0.897	0.959	0.06 (0.02–0.09)	3, 365	<0.008
CK-MB	Caspase-1	0.979	0.966	0.01 (−0.005 to –0.03)	1, 397	0.162
CK-MB	IL-1β	0.979	0.939	0,03 (0, 009–0.07)	2, 550	**0**.**01**
CK-MB	IL-18	0.979	0.959	0,02 (−0.003 to –0.04)	1, 929	0.053
Caspase-1	IL-1β	0.966	0.939	0.02 (−0, 005–0.05)	1, 620	0.105
Caspase-1	IL-18	0.966	0.959	0.007 (−0.01 to –0.03)	0, 570	0.570
IL-1β	IL-18	0.939	0.959	0.01 (−0.01 to –0.05)	1, 065	0.286

Values in bold denote statistically significant differences at the 0.05 level (*p* < 0.05).

The effect of different predictors on the predictability of the change in hs-cTnI level was examined in univariate analysis and by establishing a multivariate regression model with clinically significant parameters. In the model, CK-MB, GSDMD, caspase-1, and IL-18 were found to be predictive factors of hs-cTnI increase (Table 6).

**Table 6:** Linear regression analysis for the effect of different predictors on the predictability of the change in hs-cTnI level.

Parameters	Multivariate (enter method) linear regression analysis						Multivariate (forward method) linear regression analysis					
	Unstandardized B	Std. error	Standardized coefficients beta	t	95% CI	*p* value	Unstandardized B	Std. error	Standardized coefficients beta	t	95% CI	*p* value
CK-MB	0.035	.002	.455	14.992	0.031–0.040	**0**.**000**	0.035	.002	.455	14.992	0.031–0.040	0.000
IL-18	0.814	.072	.502	11.321	0.673–0.955	**0**.**000**	0.814	.072	.502	11.321	0.673–0.955	0.000
Gasdermin D	−0.347	.111	−.123	−3.116	−0.566 to –0.128	**0**.**002**	−0.347	.111	−.123	−3.116	−0.566 to –0.128	0.002
Caspase-1	0.453	.169	.114	2.674	0.120–0.785	**0**.**008**	0.453	.169	.114	2.674	0.120–0.785	0.008

Forward and enter methods were used for linear regression analysis.

Enter model: *R*: 0.870; R square: 0.757.

Forward LR model: R: 0.870; R square: 0.757.

CK-MB, creatine kinase-MB; IL, interleukin.

Values in bold denote statistically significant differences at the 0.05 level (*p* < 0.05).

## Discussion

Biomarkers are indispensable noninvasive tools in the diagnosis of cardiovascular diseases due to their widespread availability, ease of sampling, and relatively low cost. While classical markers such as hs-cTnI and CK-MB remain the cornerstone of AMI diagnosis, there is increasing recognition that inflammation plays a critical role in disease onset and progression. However, inflammation-related biomarkers, particularly those linked to inflammasome activation, have not been extensively investigated in AMI. In this study, we demonstrated that circulating inflammasome-related proteins, including NLRP3, GSDMD, caspase-1, IL-1β, and IL-18, were significantly elevated in STEMI patients compared with NSTEMI and controls. Among these, caspase-1, IL-18, and CK-MB exhibited superior diagnostic accuracy compared with hs-cTnI, suggesting that inflammasome-related markers could complement traditional biomarkers in the differential diagnosis of STEMI and NSTEMI. Adjusted analyses confirmed that IL-1β, GSDMD, caspase-1, and NLRP3 remained independently elevated in STEMI, even after controlling for fasting glucose, HbA1c, and smoking status, indicating that inflammasome activity contributes to STEMI pathophysiology beyond metabolic dysregulation.

NT-ProBNP, which is recommended for use in the diagnosis of heart failure in emergency departments (7), has been shown to increase after MI and can be used to decide the risk of adverse cardiovascular events (8). NT-proBNP, an established marker for heart failure, was higher in STEMI than NSTEMI in our cohort, consistent with prior studies showing its prognostic value post-MI (9–13). In the current study, pro-BNP levels were higher in the STEMI group compared to the NSTEMI. NT-proBNP levels may provide valuable information in predicting adverse major cardiac events.

All novel risk-stratification approaches using hs-cTn assays are validated according to the Universal Definition of Myocardial Infarction (UDMI) (14). Peak CK-MB and TnI levels are independently associated with in-hospital mortality, with CK-MB adding slightly more incremental prognostic value than TnI (15). STEMI and NSTEMI patients show different cTnI release patterns influenced by symptom onset, age, and CAD history, highlighting the importance of MI-type-specific evaluation to avoid biased prognostic information (16). In our study, hs-cTnI and CK-MB levels were higher in STEMI patients. ROC analysis showed that hs-cTnI (cut-off 0.09) achieved 87% sensitivity and 92% specificity, while CK-MB (cut-off 21.9) had 91% sensitivity and 94% specificity. CK-MB, caspase-1, and IL-18 demonstrated superior diagnostic accuracy compared to hs-cTnI, while IL-1β showed a higher but not statistically significant AUC. High AUC values for caspase-1, IL-1β, and IL-18 emphasize the potential diagnostic role of inflammatory processes. Incorporating inflammasome-related markers alongside classical cardiac biomarkers may enhance diagnostic accuracy and provide a more comprehensive assessment, especially in early or minimal myocardial damage (17–19).

Although NT-ProBNP, troponins, and CK-MB are routinely used in ACS, they are not elevated during the first hours of MI, limiting their utility for early diagnosis. Conventional markers require serial measurements, which is time-consuming, highlighting the need for early-stage biomarkers (20, 21). Pyroptosis, dependent on inflammatory caspases (caspase-1, 4/5/11), contributes to cell death in inflammatory diseases, cardiovascular disorders, CNS diseases, and tumors (22–25).

In our study, pyroptosis-related biomarkers, including NLRP3, IL-1β, and IL-18, were significantly higher in STEMI patients compared to NSTEMI and controls. IL-18 achieved 94% sensitivity and 87% specificity, suggesting its potential for early AMI detection. The NLRP3 inflammasome, a multiprotein complex activating IL-1β and IL-18, may provide a mechanistic link to the MI cytokine hypothesis. Nonimmune cardiac injury, such as MI, can trigger inflammation, and several studies support the role of NLRP3 in atherosclerosis and ACS. Grebe et al. (26) highlighted the contribution of the NLRP3/IL-1 pathway to vascular inflammation and plaque instability. Xu et al. (27) described pyroptosis as a driver of atherosclerotic progression. Du et al. (28) identified AIM2 and NLRP3 inflammasomes as potential diagnostic and therapeutic targets, while Gao et al. (29) reported higher plasma IL-18 in STEMI patients. Satoh et al. (30) showed elevated NLRP3, IL-1β, and IL-18 in CAD patients, with modulation by statin therapy. These findings align with our results, emphasizing the role of IL-1β and IL-18 in atherosclerosis and acute myocardial injury (26–30).

The NLRP3 inflammasome regulates caspase-1 activation, inducing pyroptosis and the release of proinflammatory cytokines IL-1β and IL-18 (31, 32). Although the regulatory mechanisms of NLRP3 are not fully understood, our study found elevated circulating inflammasome-related markers in STEMI patients, including higher plasma GSDMD and caspase-1, compared to NSTEMI. Caspase-1 demonstrated high diagnostic performance, with a cut-off value of 13 yielding 96% sensitivity and 81% specificity.

MI and MI/R-induced pyroptosis involves NLRP3-mediated caspase-1 activation, which cleaves GSDMD into GSDMD-N and GSDMD-C. GSDMD-N forms membrane pores, triggering pyroptotic cell death and releasing IL-1β and IL-18, amplifying inflammation (33, 34). Both experimental and clinical studies suggest that serum and cardiac GSDMD may serve as biomarkers for pyroptosis in MI or MI/R injury (35, 36). Elevated IL-1β and IL-18 levels in cardiomyocytes further support this mechanism (36–38).

Although the long-term prognostic value of IL-18 in STEMI remains uncertain, previous studies have shown higher serum caspase-1, GSDMD, IL-1β, and IL-18 in patients with chronic MI compared to healthy controls (35, 37). Other studies reported no significant changes in serum GSDMD after percutaneous coronary intervention in acute STEMI vs. stable CAD patients (35). Overall, NLRP3/caspase-1-mediated pyroptosis represents a key pathway of cardiomyocyte death in hypoxic cardiac tissue (39).

No significant associations were found between circulating inflammasome-related markers and hs-cTnI in STEMI patients. Univariate and multivariate regression analyses identified CK-MB, GSDMD, caspase-1, and IL-18 as predictors of TnI elevation. The lack of correlation with TnI may reflect the complex role of inflammatory mediators in both adaptive and maladaptive responses during MI, with the relative contribution of individual factors remaining unclear (40).

Studies on the NLRP3 inflammasome in MI and myocardial I/R injury highlight unresolved questions. As these proteins are involved in systemic inflammatory responses, the circulating amounts released during MI may be too low to directly impact total plasma levels. Nevertheless, these findings support targeting the NLRP3 inflammasome pathway therapeutically, potentially optimizing the timing and specificity of anti-inflammatory interventions (41). Given its role in atherosclerosis development, the NLRP3 inflammasome remains a promising therapeutic target, with novel inhibitors under investigation. Moreover, NLRP3 activation, caspase-1–mediated pyroptosis, and IL-1β/IL-18–driven inflammation are key contributors to ischemia-reperfusion injury following acute MI (32, 42).

### The study of limitations

This study has several limitations. It was conducted at a single center, which may limit generalizability, and only baseline biomarker levels were measured, preventing assessment of temporal changes. Although adjustments were made for glucose, HbA1c, and smoking, other potential confounders were not evaluated. The relatively small sample size and lack of external validation further limit the robustness of the findings. Long-term clinical outcomes using inflammasome markers could not be assessed. Larger multicenter studies with longitudinal follow-up are needed to confirm these results and determine their prognostic implications.

In the initial patient assessment, a negative hs-cTnI alone cannot rule out ACS, although hs-cTnI remains the only biomarker currently recommended for AMI diagnosis due to its superior sensitivity and accuracy. Our findings suggest that the NLRP3 inflammasome plays a key role in producing pro-inflammatory cytokines such as IL-1β and IL-18 in STEMI, and caspase-1–mediated pyroptosis is the predominant pathway following STEMI. CK-MB, caspase-1, and IL-18 demonstrated superior diagnostic accuracy compared with hs-cTnI, indicating their potential as valuable adjunct biomarkers. Pyroptosis-related markers, particularly IL-1β and IL-18, may aid in early diagnosis and risk stratification, especially in complex or ambiguous cases, and understanding inflammasome pathways could inform targeted anti-inflammatory therapies in MI management. The study also highlights the NLRP3 inflammasome as a potential therapeutic target due to its role in atherosclerosis. While our sample size was determined by patient availability rather than *a priori* calculation, *post hoc* power analysis indicated over 80% power, suggesting sufficient reliability. Further research is needed to validate these findings across diverse clinical settings and to address remaining mechanistic questions.

## Data Availability

The original contributions presented in the study are included in the article/Supplementary Material, further inquiries can be directed to the corresponding author.
